# A New Glycan-Dependent CD4-Binding Site Neutralizing Antibody Exerts Pressure on HIV-1 *In Vivo*

**DOI:** 10.1371/journal.ppat.1005238

**Published:** 2015-10-30

**Authors:** Natalia T. Freund, Joshua A. Horwitz, Lilian Nogueira, Stuart A. Sievers, Louise Scharf, Johannes F. Scheid, Anna Gazumyan, Cassie Liu, Klara Velinzon, Ariel Goldenthal, Rogier W. Sanders, John P. Moore, Pamela J. Bjorkman, Michael S. Seaman, Bruce D. Walker, Florian Klein, Michel C. Nussenzweig

**Affiliations:** 1 Laboratory of Molecular Immunology, The Rockefeller University, New York, New York, United States of America; 2 Division of Biology and Biological Engineering, California Institute of Technology, Pasadena, California, United States of America; 3 Ragon Institute of MGH, MIT and Harvard, Cambridge, Massachusetts, United States of America; 4 Department of Medical Microbiology, Academic Medical Center, University of Amsterdam, Amsterdam, The Netherlands; 5 Department of Microbiology and Immunology, Weill Medical College, Cornell University, New York, New York, United States of America; 6 Center for Virology and Vaccine Research, Beth Israel Deaconess Medical Center, Harvard Medical School, Boston, Massachusetts, United States of America; 7 Howard Hughes Medical Institute, Chevy Chase, Maryland, United States of America; 8 First Department of Internal Medicine, University Hospital of Cologne, Cologne, Germany; 9 Center for Molecular Medicine Cologne (CMMC), University of Cologne, Germany; Miller School of Medicine, UNITED STATES

## Abstract

The CD4 binding site (CD4bs) on the envelope glycoprotein is a major site of vulnerability that is conserved among different HIV-1 isolates. Many broadly neutralizing antibodies (bNAbs) to the CD4bs belong to the VRC01 class, sharing highly restricted origins, recognition mechanisms and viral escape pathways. We sought to isolate new anti-CD4bs bNAbs with different origins and mechanisms of action. Using a gp120 2CC core as bait, we isolated antibodies encoded by IGVH3-21 and IGVL3-1 genes with long CDRH3s that depend on the presence of the N-linked glycan at position-276 for activity. This binding mode is similar to the previously identified antibody HJ16, however the new antibodies identified herein are more potent and broad. The most potent variant, 179NC75, had a geometric mean IC_80_ value of 0.42 μg/ml against 120 Tier-2 HIV-1 pseudoviruses in the TZM.bl assay. Although this group of CD4bs glycan-dependent antibodies can be broadly and potently neutralizing *in vitro*, their *in vivo* activity has not been tested to date. Here, we report that 179NC75 is highly active when administered to HIV-1-infected humanized mice, where it selects for escape variants that lack a glycan site at position-276. The same glycan was absent from the virus isolated from the 179NC75 donor, implying that the antibody also exerts selection pressure in humans.

## Author Summary

CD4bs is a central viral vulnerability site and isolation of new anti-HIV-1 CD4bs broadly neutralizing antibodies (bNAbs) provides information about viral escape mechanisms. Here we describe a new anti-HIV-1 bNAb that was isolated from an HIV-1 infected donor. The antibody, 179NC75, targets the CD4 binding site in a glycan-dependent manner. Although many CD4bs antibodies have been already described, a glycan-dependent mode of recognition is unusual for anti-HIV-1 CD4bs bNAbs. The glycan-dependent CD4bs antibodies have never been tested for their ability to neutralize HIV-1 *in vivo*. We infected humanized mice with HIV-1_YU2_ and treated them with 179NC75 three weeks after infection. We observed a drop in viral load immediately after treatment followed by a viral rebound. The viral rebound was associated with specific escape mutations in the plasma virus envelope, resulting in a deletion of N276 glycan, and in some cases a glycan shift from position 276 to position 460. Similar signature mutations were found in the envelope of the autologous virus cloned from patient’s plasma. This defines the escape pathways from 179NC75, and shows that they are the same in humans and in HIV-1_YU2_ infected humanized mice.

## Introduction

Although the envelope glycoproteins (Env) of primate immunodeficiency viruses have extremely variable sequences [1], most of them engage CD4 as the primary cellular receptor to initiate the viral life cycle [2]. The consequence is that the CD4 binding site (CD4bs) is a comparatively well-conserved region of Env that serves as a critical neutralization epitope and an appealing vaccine target. The introduction of single cell antibody cloning techniques [3,4] yielded dozens of broad and potent CD4bs antibodies from infected individuals, some of which neutralize ~90% of HIV-1 strains *in vitro* [5–7]. Some of these antibodies are also effective at reducing viral load when used to treat infected humanized mice (hu-mice) [8], macaques [9–11] and humans [12].

The most potent group of CD4bs antibodies characterized to date is derived from two VH genes, IGVH1-2 [5,7,13] and IGVH1-46 [6,7,14–16]. These antibodies engage many of the same Env residues as CD4. For example, residue Arg71_HC_ in VRC01-like bNAbs interacts with residue Asp368_gp120_ on Env, and thereby mimics how Arg59_CD4_ interacts with the same residue when CD4 binds to gp120 [6,7,13,16]. Although the light chains are less restricted in their origin, specific alterations are required for activity, including mutations and deletions [6,13,16]. Overall, the restricted origins and complex development of these bNAbs from their inactive germline precursors may explain why it has been so difficult to elicit them by vaccination.

A second, far more diverse group of CD4bs-directed antibodies is often referred to as ‘CDRH3-dominated class of CD4bs antibodies’. These antibodies use their CDRH3-loop regions to engage Env [15]. These include b12 [17], HJ16 [18], CH103 [19] and the recently described VRC13 and VRC16 [15]. Structural analyses indicate that all CDRH3-dominated antibodies use loop-based recognition mechanisms, with the CDRH3 contributing 50%-70% of the paratope interface [15,19,20]. They are not VH-restricted since their CDRH3s are randomly assembled from IgH variable, diversity and joining segments during V(D)J recombination [21]. In keeping with their diverse origins, CDRH3-dominated antibodies seem to employ different mechanisms of recognition and they also vary in the angles with which they approach the CD4bs [15].

To isolate new CD4bs bNAbs, we sought HIV-1 infected donors whose sera contained potent neutralizing antibodies that appeared to target the CD4bs. One such donor was EB179. By sorting peripheral blood mononuclear cells (PBMCs) from this individual we isolated a new antibody, 179NC75, that is encoded by IGVH3-21 and IGVL3-1 gene segments. In TZM.bl neutralization assays 179NC75 showed an overall IC_80_ of 0.42 μg/ml against 120 Tier-2 HIV-1.

Binding assays using various Env-based proteins indicated that 179NC75 is glycan-dependent and belongs to the same sub-class of CDRH3-dominated CD4bs antibodies as HJ16. These glycan-dependent CD4bs antibodies have not yet been tested for activity *in vivo*. To do so we treated humanized mice infected with HIV-1_YU2_ with 179NC75 and found that it selects for escape variants with mutations in the potential N-linked glycosylation site at gp120 position 276. Similar mutations were also found in the autologous isolate from the 179NC75 donor, suggesting that selection pressure had been exerted in the human host.

## Materials and Methods

### Ethics statement

For the human studies, The Rockefeller University Institutional Review Board approved all studies involving patient enrollment, sample collection, and clinical follow-up. Donor EB179 was selected from a group of long-term non- progressors that was followed at the Ragon Institute in Boston, and is also referred to as subject 330183. The subject described in this study provided written informed consent prior to participating in this study. For the mouse studies, this study was carried out in strict accordance with the recommendations in the Guide for the Care and Use of Laboratory Animals of the National Institutes of Health. The protocol was approved by the Institutional Animal Care and Use Committee (IACUC) of The Rockefeller University, and in accordance with established guidelines and policies at The Rockefeller University (protocol number 13618-H).

### HIV-1-infected subjects

Purified IgG samples from 394 HIV-1-infected long-term non-progressors were screened for neutralizing activity against a panel of 14 viruses representing 8 different clades or inter-clade recombinants. IgG purified from donor EB179 was exceptional in its neutralization potency and breadth, ranking within the top 2% of the cohort, and neutralized 11 out of the 14 viruses in the panel (S1A Table). A single leukapheresis sample was obtained 4.5 years after initial diagnosis with clade B HIV-1 infection, at age 44. At the time the sample was collected, donor EB179 had 1038 CD4 T cells/mm^3^ and a viral load of 3180 copies/ml and was not receiving antiretroviral therapy. Molecular HLA typing revealed HLA A*02:01, 68:02; B*07:02, 53:01; Cw04:01, 07:02; DRB11:01, 15:01.

### Single B cell sorting and antibody cloning

Single-cell sorting of 2CC core^+^CD19^+^IgG^+^ B cells from donor EB179’s PBMCs was conducted as previously described [3]. Briefly, we sorted memory B cells using the gp120 2CC core protein as bait [22]. Rescue primers were used to amplify both heavy chains [7] and Igλ genes [23]. All PCR products were sequenced and analyzed for Ig gene usage, CDR3, and the number of VH/VL somatic hypermutations (IgBLAST, http://www.ncbi.nlm.nih.gov/igblast/ and IMGT, http://www.imgt.org/). Multiple sequence alignments were performed using the MacVector program (v.13.5.5) with the ClustalW analysis function (default parameters), and then used to generate dendrograms by the neighbor-joining method (with best tree mode and outgroup rooting). To specifically isolate members of the 179NC75 clone we used the following forward primers for the heavy chains: 5’-CTGCAACCGGTGTACATTCTGAAATGAGATTGGAAGAAT-3’ and 5’-CTGCAACCGGTGTACATTCTGAGGTCCAGTGTGAAGAA-3’ (in a 1:1 mix); and for the light chains: 5’-ATGGCCTGGATCCCTCTACTTCTC-3’ and 5’- ATGGCATGGATCCCTCTCTTCCTC-3’ (in a 1:1 mix). The reverse primers were the same as described previously for Ig gene amplification [7].

### Antibody production

Purified, digested PCR products were cloned into human Igγ1-, IgK or Igλ-expression vectors as previously described [24]. Antibodies were produced by transient transfection of IgH, IgK and IgL expression plasmids into exponentially growing HEK 293T cells (ATCC; CRL-11268) using polyethyleneimine (PEI)-precipitation [24]. IgG antibodies were affinity purified using Protein G Sepharose beads according to the manufacturer’s instructions (GE Healthcare).

### ELISAs

High-binding 96-well ELISA plates (Costar) were coated overnight with 5 μg/ml of purified 2CC core, gp120.YU2 (wild type or mutants) or gp140.YU2 foldon trimer in PBS. After washing 6 times with PBS + 0.05% Tween 20, the plates were blocked for 2 h with 2% BSA, 1 μM EDTA and 0.05% Tween-PBS (“blocking buffer”), and then incubated for 1 h with IgGs that were added as seven consecutive 1:4 dilutions in PBS from an initial concentration of 4 μg/ml. After additional washing, the plates were developed by incubation with goat HRP-conjugated anti-human IgG antibodies (Jackson ImmunoResearch) (at 0.8 μg/ml in blocking buffer) for 1 h followed by HRP chromogenic substrate (ABTS solution; Invitrogen). For competition ELISAs, the plates were coated with 5 μg/ml 2CC core, gp120 or gp140 foldon, washed, blocked for 2 h with blocking buffer and then incubated for 1 h with IgGs added as seven consecutive 1:4 dilutions in PBS from an initial concentration of 32 μg/ml, and in the presence of biotinylated 179NC75 antibody at a constant concentration of 4 μg/ml. The plates were then developed using HRP-congugated streptavidin (Jackson ImmunoReseach) (at 1 μg/ml in blocking buffer).

For ELISAs using BG505 SOSIP.664-D7324 trimers, the plates were coated overnight with 5 μg/ml of D7324 antibody as previously described [25], washed and then incubated with 500 ng/ml of the trimer [25,26]. After a further wash, IgGs were added for 1 h as seven consecutive 1:4 dilutions in PBS from initial concentrations of 4 μg/ml. The endpoint was generated by incubation with goat HRP-conjugated anti-human IgG antibodies, as described above. All experiments were performed at least 3 times.

For EndoH ELISA, the plates were coated overnight at 4°C with 5 μg/ml of EndoH-treated or untreated gp120 in 100 mM sodium bicarbonate/carbonate buffer, pH 9.6. They were then washed with TBS + 0.05% Tween 20 and blocked for 1 h in the same buffer supplemented with 3% (w/v) BSA, and washed again before test antibodies were added for 2 h. After a final wash, the endpoint was generated using goat HRP-conjugated anti-human IgG antibodies, again as described above.

### Neutralization assay

HIV-1 neutralization was evaluated using the luciferase-based TZM.bl cell assay as described previously [27]. Briefly, envelope pseudoviruses were incubated with fivefold serial dilutions of single antibodies and applied to TZM.bl cells that carry a luciferase-reporter gene. After 48 h cells were lysed and luminescence was measured. IC_50_ and IC_80_ reflect single antibody concentrations that caused a reduction in relative luminescence units (RLU) by 50% and 80%, respectively.

### Humanized mice (hu-mice)

NOD *Rag1* ^−/−^ *Il2rg* ^null^ (NOD.Cg-*Rag1* ^*tm1Mom*^ *Il2rg* ^*tm1Wjl*^/SzJ) mice were purchased from The Jackson Laboratory and bred and maintained at the Comparative Bioscience Center of The Rockefeller University according to guidelines established by the University’s Institutional Animal Care and Use Committee. All experiments were performed under protocols approved by the same committee. Hu-mice were treated with 1 mg of 179NC75 sub-cutaneously (s.c.) on day 0, followed by 0.5 mg s.c. injections twice-weekly for a period of 5 weeks [8]. The gp120 sequences from escape variant viruses were obtained as previously described [8].

### Virus cloning and virus co-culture

The autologous virus from donor EB179 was isolated as previously described [28]. Briefly, CD19 and CD8-depleted mononuclear cells were cultured at a concentration of 5 × 10^6^ cells/ml in Iscove's modified Dulbecco’s medium (IMDM; Gibco) supplemented with 10% fetal bovine serum (FBS; HyClone, Thermo Scientific), 1% GlutaMAX (Gibco), 1% penicillin/streptomycin (Gibco), and 1 μg/ml phytohaemagglutinin (Life Technologies) at 37°C and in an atmosphere containing 5% CO_2_. After 2–3 days, 5 × 10^6^ cells were transferred into IMDM supplemented with 10% FBS, 1% penicillin/streptomycin, 5 μg/ml polybrene (Sigma), and 100 IU/ml of IL-2. The medium was replaced weekly and the HIV-1 content of culture supernatants was quantified using the Lenti-X p24 Rapid Titer Kit (Clontech) according to the manufacturer’s instructions. *Env* genes from the autologous virus were cloned by reverse transcriptase PCR as described elsewhere [29].

### HIV-1_YU2_ envelope mutants

Single, double and triple mutations were introduced into wild-type HIV-1_YU2_ envelope using the QuikChange (multi-) site-directed mutagenesis kit, according to the manufacturer’s specifications (Agilent Technologies).

## Results

### Serologic specificity

Polyclonal IgG purified from donor EB179 had exceptional neutralization capacity, with respect of potency and activity against 11 of 14 Tier-2 viruses in a small cross-clade panel (S1A Table). To map the predominant NAb specificities, we tested EB179 IgG against HIV-1_YU2_ mutants that are resistant to NAbs targeting the trimer apex (N160K), the CD4bs (N280Y) or the base of the V3 loop (N332K) [8,30–32]. Among these mutants, only HIV_YU2_N280Y was resistant to EB179 IgG (S1B Table). We conclude that at least a proportion of the neutralization activity present in this serum is directed to the CD4bs.

### EB179 CD4bs antibody repertoire

**Fig 1:**
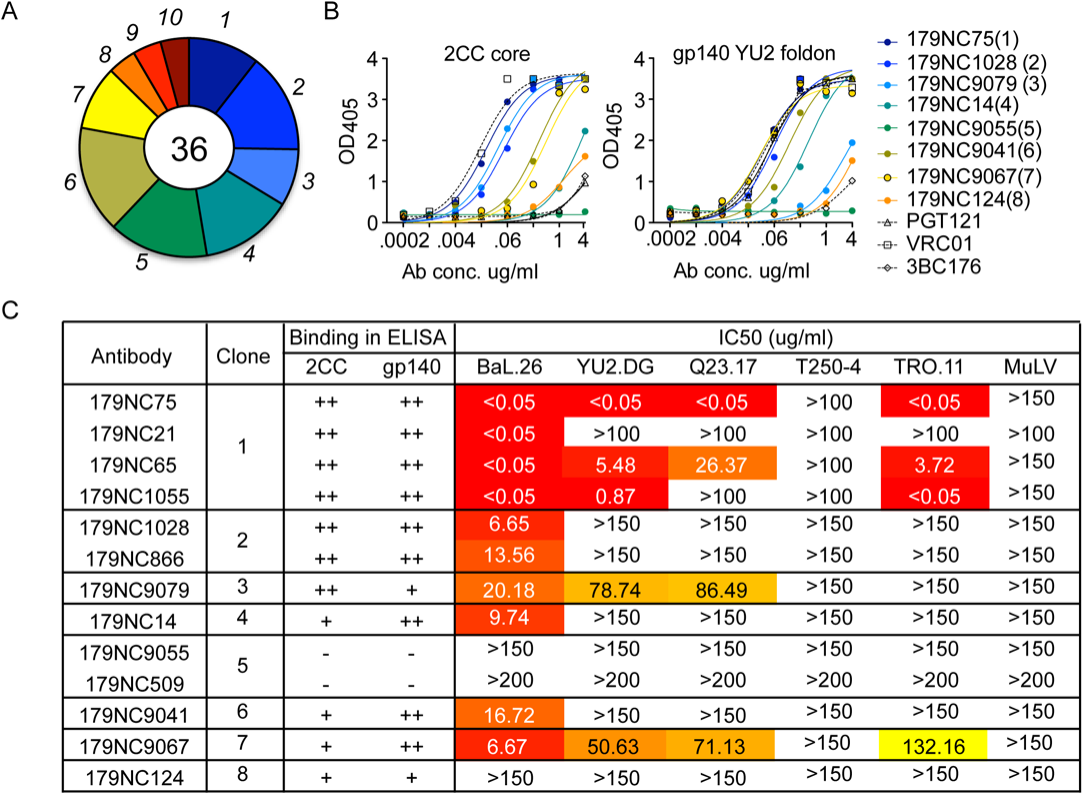
2CC core antibody repertoire in patient EB179. (A) Pie chart representing ten antibody sequence families sorted using the 2CC core protein as bait. The number in the center of the pie denotes the number of antibodies; slices are proportional to clone size (i.e., frequency) and represent unique clones. The assigned number of each clone is given next to the corresponding slice on the pie chart. The membership of an antibody in a B cell clone is determined by sequence analysis, in particular of the CDR3s and shared V and J genes of paired heavy and light chain genes (see Methods). (B) Representative variants of each clonal family of sequences were expressed and tested in ELISA for binding to 2CC core and gp140 foldon proteins. The numbers in parentheses correspond to the number of the clone as shown in (A). (C) The table summarizes the different antibodies expressed, the clone they represent and their binding to 2CC core or gp140 YU2 foldon (based on (B)) and their *in vitro* neutralization IC_50_ values, as measured in the TZM.bl cell assay.

To isolate and characterize the NAbs present in EB179, we used flow cytometry to sort memory B cells that bound to 2CC core, a gp120 antigen that presents the CD4bs in an exposed and stable conformation [22]. Among CD19^+^IgG^+^ B cells, ~0.2% bound strongly to 2CC core. Of the 372 cells sorted, 87 produced paired heavy and light chains, 36 of which represented ten clonally related families (Fig 1A). Antibody sequences obtained from the expanded B cell clones contained higher numbers of somatic mutations compared to antibodies obtained from B cells that appeared only once (S1 Fig). The average number of nucleotide mutations in the heavy chain of clonal sequences was 44.76 (± 3.66, N = 36) compared to 20.82 (± 1.39 N = 51) for unique sequences (S1A Fig). A similar trend was observed when the light chain sequences were analyzed (S1B Fig).

Representative variants from each of the clonal families were selected for further analysis (S2 Table). These variants were expressed as IgG1 antibodies that were tested for binding to a HIV-1_YU2_ gp140 foldon protein [33] or 2CC core [22], and for neutralizing activity. Except for 179NC9055, all the antibodies bound strongly to the HIV-1_YU2_ gp140 and/or 2CC core proteins (Fig 1B), and members of clones 1, 2, 3, 4, 6, and 7 neutralized the Tier-1 (i.e., neutralization-sensitive) HIV-1_BAL_ virus (Fig 1C). While antibodies from clones 3 and 7 were only weakly active against the other viruses in the panel, one representative of the most expanded clone 1 (179NC75) strongly neutralized four of the five viruses tested (IC_50_ ≤0.05 μg/ml, Fig 1C).

### 179NC75 and its clonal relatives

**Fig 2:**
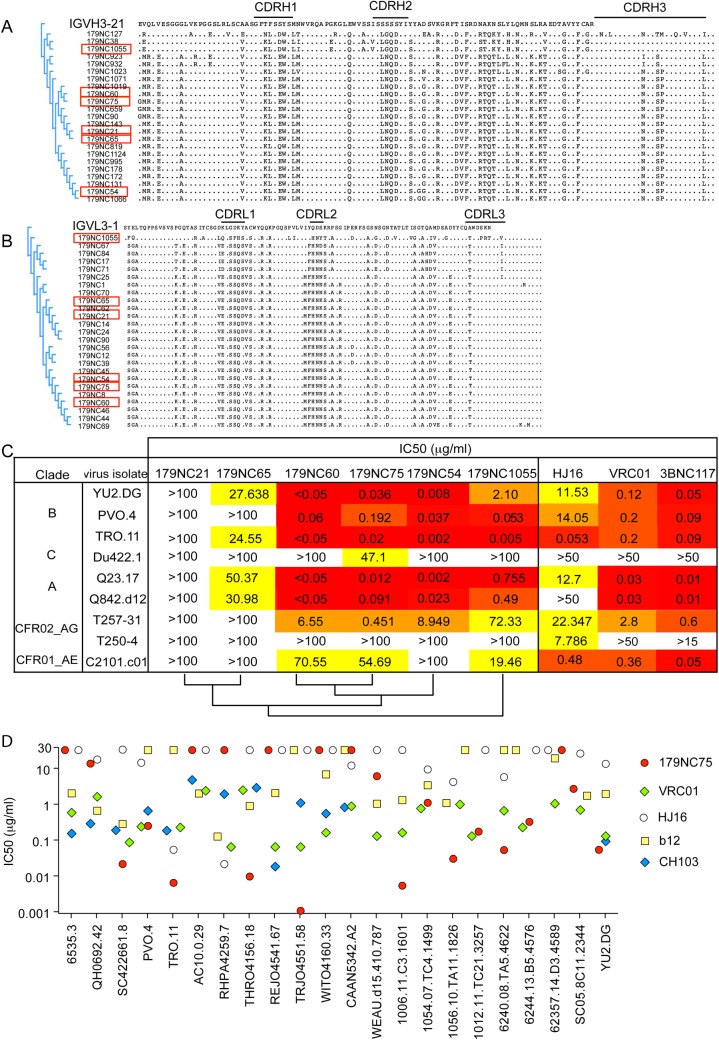
179NC75 clonal family variants. (A) Dendrogram showing heavy chain amino acid sequence alignment of the different 179NC75 variants to their closest germline IGVH3-21. (B) The same plot as in (A), but for the light chains. The heavy and light chains of the variants that were expressed and tested for neutralization are marked with red squares. (C) Neutralization IC_50_ values for 179NC75 and five variants against an expanded panel of Tier-2 viruses. The phylogenetic relationship, based on heavy chain sequences, between the six variants are shown on the bottom of the table (D) The plot compares the IC_50_ values for 179NC75 (red circles) and four well characterized CD4bs bNAbs (VRC01 –green diamonds, b12 –yellow squares, CH103 –blue diamonds and HJ16 –white circles) against a panel of 26 clade B Tier-2 viruses.

To isolate additional 179NC75 variants, we amplified cDNA from the 2CC core^+^CD19^+^IgG^+^ single-sorted B cells using specific VH and VL forward primers (see Methods). We obtained a total of 23 heavy chain and 25 light chain variants from the 179NC75 clonal family. The heavy and light chain sequences carried 34% and 29% amino acid mutations on average, respectively, compared to their germline gene segments IGVH3-21 and IGVL3-1. The various sequences of the 179NC75 clone were similar by up to 73% from clonal members (Fig 2A and 2B). The CDRH3 and CDRL3 regions were 24 and 10 residues long, respectively (Fig 2A and 2B, S2 Table). There were no insertions or deletions.

Variants 179NC 54, 60, 65, 75, 21 and 1055 (indicated in Fig 2A and 2B) were tested for activity against a panel of nine Tier-2 viruses, including three from clade B, one from clade C, two from clade A, two clade A/G recombinants and one clade A/E recombinant. 179NC75 and two closely related variants, 179NC54 and 179NC60, potently neutralized 6 of these 9 viruses, whereas the other antibodies had lesser or no neutralization activity (Fig 2C). Accordingly, we selected 179NC75 for additional analyses.

When tested against an extended cross-clade panel of 120 Tier-2 viruses, 179NC75 neutralized viruses from clades B particularly strongly (S3 Table); its geometric mean IC_50_ and IC_80_ values were 0.113 μg/ml and 0.291 μg/ml, respectively (S4 Table). When compared to other CD4bs bNAbs against a panel of 22 Tier-2 clade, B viruses, 179NC75 was more potent than b12 against 13 viruses, than HJ16 against 15 viruses, than VRC01 against 8 viruses, and than CH103 against 6 viruses (Fig 2D). Its overall breadth of activity across the clade B virus panel was 70% (S4 Table).

### 179NC75 binds Env by an HJ16-type mechanism

**Fig 3:**
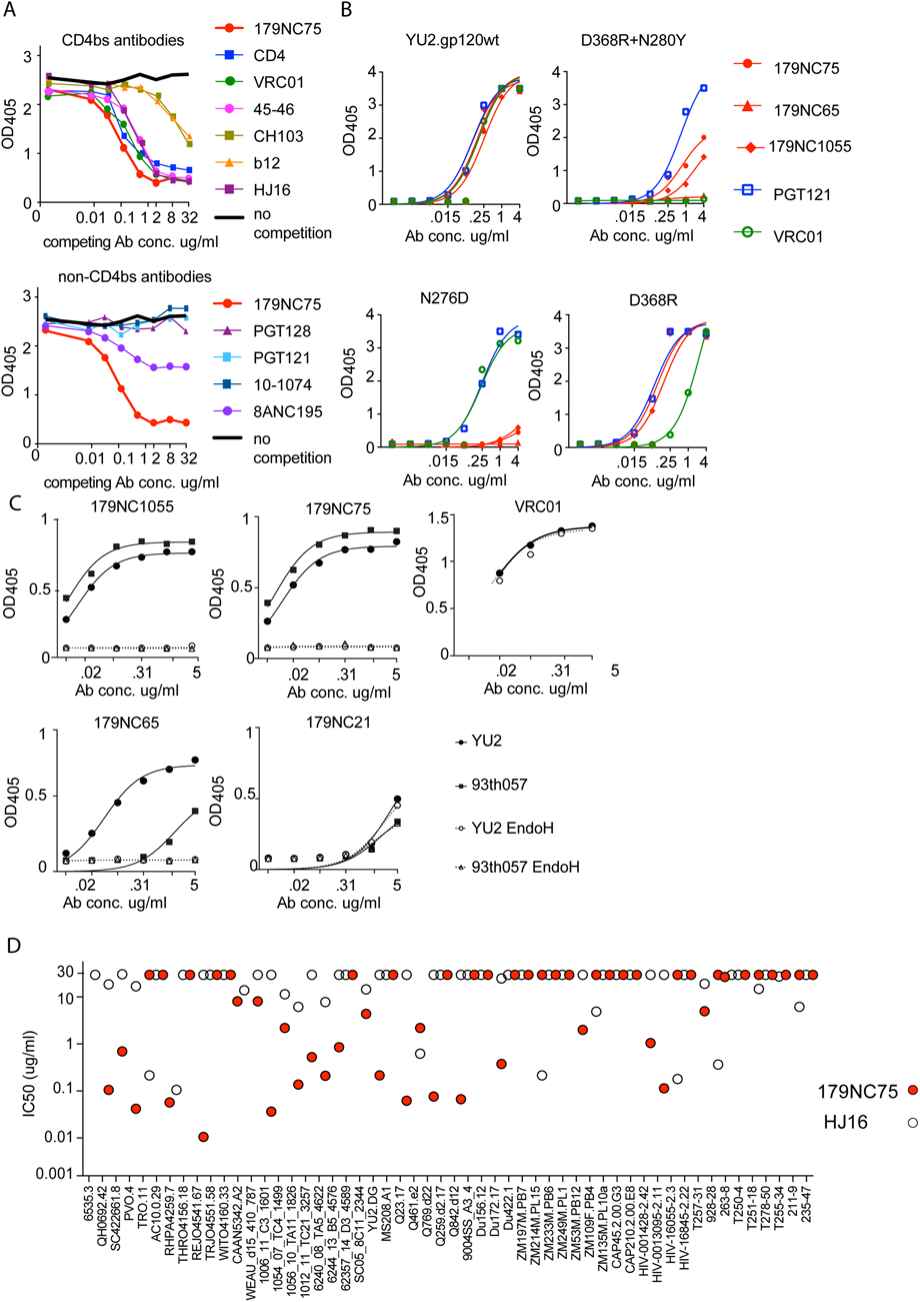
Epitope mapping of 179NC75 variants by ELISA. (A) Competition ELISA. The plots show the binding of biotinylated 179NC75 (constant concentration of 2 μg/ml) to gp120 in the presence of increasing concentrations of different bNAbs. The black bold line represents 179NC75 binding in the absence of a competitor antibody, the red bold line shows auto-competition by non-biotinylated 179NC75. Upper panel: Competition by CD4bs bNAbs; lower panel competition by non-CD4bs bNAbs. (B) Binding of 179NC75 (red circles), 179NC65 (red triangles) and 179NC1055 (red diamonds) antibodies to the wild-type YU2 gp120 monomer (left upper panel) and variants containing CD4bs-related mutations (D368R+N280Y, right upper panel; D368R, right lower panel; N276D, left lower panel), VRC01 and PGT121 served as control antibodies. (C) 179NC75, 179NC65 and 179NC1055 binding to gp120 monomers from YU2 (clade B) and 93TH057 (clade A/E) before and after deglycosylation with EndoH. VRC01 was used as a control antibody in the experiment involving the YU2 gp120s. (D) Comparison between the neutralizing activity (IC_50_) of 179NC75 and HJ16 against a cross-clade virus panel of Tier-2 viruses.

To map the epitope targeted by 179NC75 and its clonal variants, we performed a series of ELISAs. All members of the 179NC75 clonal family bound to HIV-1_YU2_ gp120, gp140 foldon [34] and 2CC core [22] proteins (S2 Fig). In a competition ELISA, soluble CD4 (sCD4) and most CD4bs antibodies competed with 179NC75 for binding to gp120_YU2_, whereas PGT121, PGT128 and 10–1074 did not (Fig 3A upper and lower panels). The 8ANC195 bNAb, which binds an epitope adjacent to the CD4bs [7,35], inhibited 179NC75 binding by ~ 50% (Fig 3A, lower panel). We conclude that the 179NC75 epitope is proximal to the CD4bs.

We next tested how different mutations in the CD4bs affected 179NC75 binding. The D368R single mutation was not sufficient to affect the gp120-binding of 179NC75 family members, but the D368R and N280Y double mutation substantially impaired their binding. In contrast, VRC01 is sensitive to the single D368R substitution (Fig 3B, right upper and lower panels).

The Asn276 glycan site is important for the binding of two different bNAbs: the CD4bs antibody HJ16 [36] and the gp120-gp41 specific antibody 8ANC195 [7,35]. The 8ANC195 epitope lies outside the CD4bs and this antibody binds Env in the presence of CD4 [7,35]. Since HJ16 strongly inhibited 179NC75 binding (Fig 3A, upper panel) and 8ANC195 did so weakly (Fig 3A, lower panel), we assessed whether the binding of 179NC75 family members was affected by the N276D substitution and found that it had a profound impact (Fig 3B, lower left panel). In contrast, the N276D change had no effect on VRC01 binding, as previously reported [37] (Fig 3B). When monomeric gp120s from both YU2 and the clade A/E virus 93TH057 [38] were treated with EndoH, a glycosidase that removes *N*-linked oligomannose glycans, the binding of 179NC75 and its clonal variants was completely abolished (Fig 3C). To further probe the nature of the glycan-dependency of 179NC75, we tested binding of the Fab to BG505 SOSIP.664 trimers, (fully glycosylated, cleaved, native-like, soluble trimers [25]) produced in HEK293-6E cells in the presence and in the absence of the mannosidase I inhibitor kifunensine. HEK293-6E cells fully process glycans resulting in a mixture of complex-type and high-mannose *N*-glycans, while HEK293-6E cells treated with kifunensine, produce protein containing only high-mannose *N*-glycans. We observed that 179NC75 binds to BG505 SOSIP.664 trimer with processed glycans with a *K*_D_ of ~90 nM, (S3 Fig) but cannot bind to trimers containing only high mannose glycans (S3 Fig, S6 Table). Hence, we conclude that 179NC75 is a glycan-dependent antibody that binds to the CD4bs in a way that involves the Asn276 residue and depends on the presence of complex glycans. In these respects, its epitope is similar to that of the HJ16 CD4bs bNAb.

We compared the neutralization potencies of 179NC75 to the ones of HJ16 [18]. For the 53 Tier-2 viruses that were tested against both HJ16 and 179NC75, 179NC75 neutralized more viruses than HJ16 (26 compared to 19), and was 20-fold more potent (IC_50_ of 0.118 μg/ml compared to 2.326 μg/ml) (Fig 3D).

### Predicted germline variant of 179NC75 binds to BG505 SOSIP

Previous reports show that neutralizing antibodies bind BG505 SOSIP.664 trimers with higher affinity as opposed to non-neutralizing antibodies [25]. Therefore, as expected, the more potent variants of the 179NC75 clone, 179NC75 and 179NC1055, bound strongly to BG505 SOSIP.664-D7324 trimers in capture ELISA, while 179NC65 and 179NC21 bound weakly or not at all, respectively (S4 Fig).

**Fig 4:**
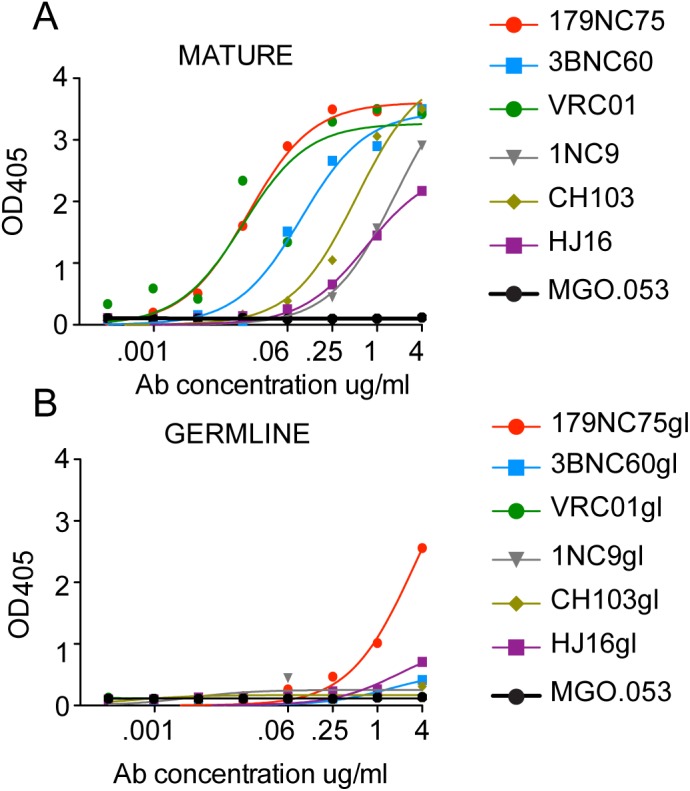
Mature *versus* predicted germline antibody binding to BG505 SOSIP.664-D7324 trimers in ELISA. (A) and (B) Binding of mutated CD4bs bNAbs (“MATURE”) and their reverted germline versions (“GERMLINE”) to BG505 SOSIP,664-D7324 trimers. The MGO.053 antibody [48] was used as a negative control.

Most predicted germline versions of CD4bs antibodies are unable to bind Env antigens [7]. To test whether the germline version of 179NC75 could bind the BG505 SOSIP.664-D7324 trimers, and assess the role of CDRH3 in trimer binding, we generated a germline version of 179NC75 (179NC75gl). The predicted germline version of the antibody was made as previously described by reverting the V and J segments of the heavy and light chains to their predicted germline sequences, while retaining the CDRH3 sequence as found in the mutated antibody [7,39,40]. For comparison, we used the previously published predicted germline versions of VRC01 [39,40], 3BNC60 [7], 1NC9 [7], CH103 [19] and HJ16 (constructed in the course of this study). Although all of the above mature CD4bs bNAbs bound the BG505 SOSIP.664-D7324 trimers, the only predicted germline antibody able to do so was 179NC75gl (Fig 4). An implication is that 179NC75 binding principally involves contacts made by the CDR3s, particularly the exceptionally long (24-residue) heavy chain CDR3.

### *In vivo* activity

**Fig 5:**
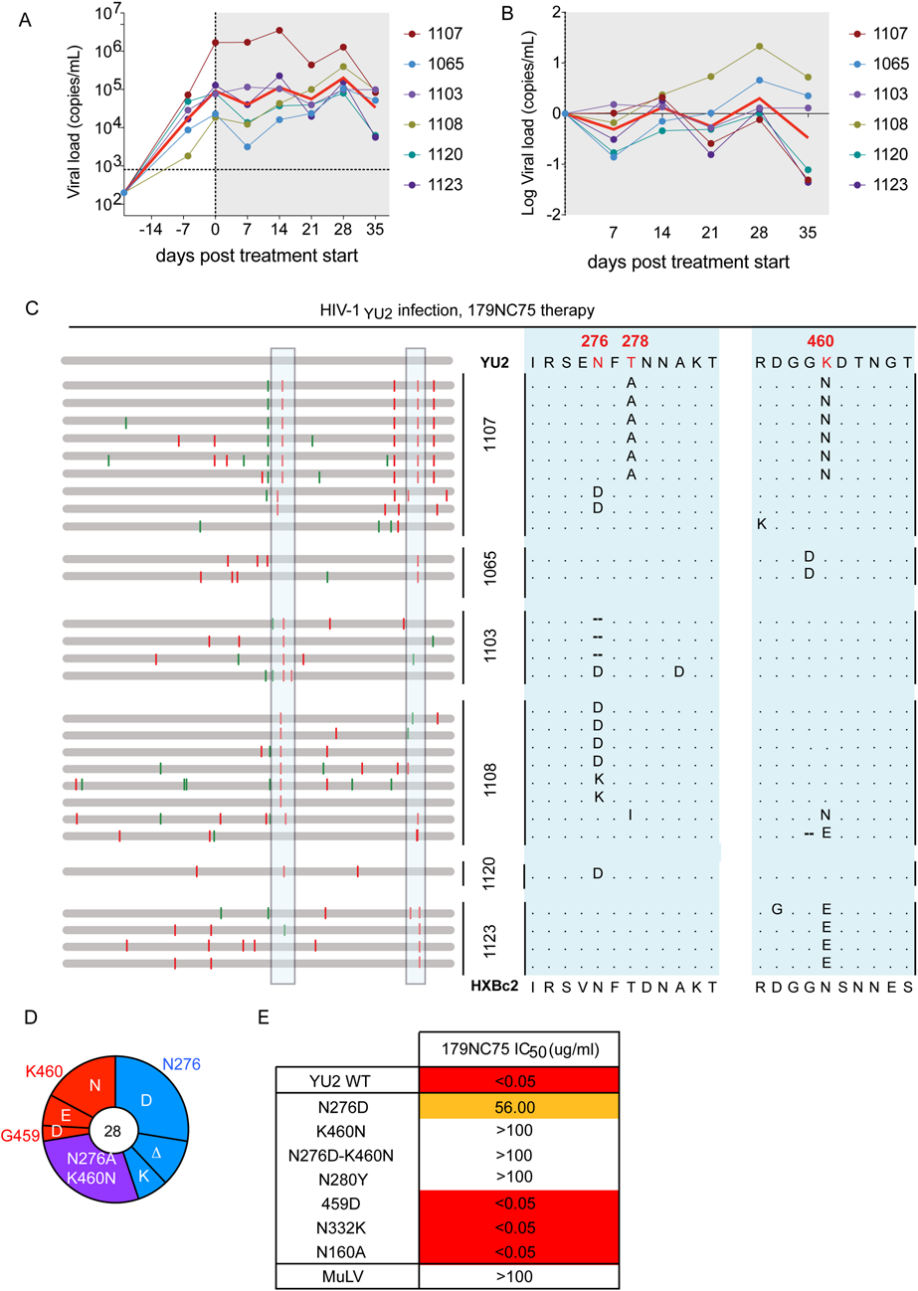
Hu-mice treatment with 179NC75. (A) Viral loads in six HIV-1_YU2_-infected hu-mice when 179NC75 was administered two weeks post-infection (day-0). The red line indicates the geometric mean value. (B) Log change in viral loads in each mouse compared to the day 0 value. (C) Left panel: each horizontal gray bar represents the sequence of a single gp120 clone aligned to HIV-1_YU2_. Synonymous and non-synonymous nucleotide substitutions are indicated in green and red, respectively. Sequences are grouped by the mouse from which they were obtained (center), indicated by the vertical black bars. An expanded view of the boxed areas is shown in the right panel. (D) Pie chart showing the recurrent mutations in gp120 compared to the wild-type HIV-1_YU2_ sequence. The number in the center of the pie denotes the total number of sequences cloned; the blue, red and purple slices represent the most consistently mutated areas in gp120, i.e., around residues 276 and 460, respectively. The sizes of the slices are proportional to the number of sequences that carried mutations. The original residue, as found in HIV-1_YU2,_ is indicated on the outside of the pie chart, the various mutations in the cloned sequences are shown on the inside of the chart. “Δ” denotes a deletion of the residue. The purple slice indicates the presence of both the blue (276-area) and red (460-area) mutations. (E) The table shows IC_50_ values for 179NC75 neutralization of the wild-type HIV-1_YU2_ pseudovirus and mutants containing either the gp120 changes most frequently observed after treatment or other mutations that serve as comparators.

The loop binding, glycan-dependent CD4bs bNAbs have not been tested for their activity *in vivo*. To address this issue, we treated six HIV-1_YU2_–infected hu-mice with 179NC75 for 5 weeks [8,29]. Monotherapy with 179NC75 resembled monotherapy with other bNAbs, in that there was a transient decrease in viral load in most of the treated animals followed by a rapid rebound [8,29,41] (Fig 5A and 5B). Viral *env* genes were cloned and sequenced from the day-28 plasma of 179NC75-treated mice, a time point where viremia had universally rebounded to levels similar to the day-0 value. Two types of mutations were consistently observed, both proximal to the CD4bs: the first eliminated the glycan-site at position N276; the second involved residues G459 or K460 (Fig 5C). In total, 13 sequences had only a mutation affecting the N276 glycan site, whereas 8 contained mutations in the region near position 460 and 7 sequences contained mutations in both regions (Fig 5C and 5D). In all mice the rebounding viruses carried at least one of these mutations. In mouse 1107 mutations in both areas were observed, resulting in the loss of the N276 glycan but the introduction of a potential N-linked glycosylation site (PNGS) at position 460 (Fig 5C). To confirm that the most commonly observed mutations did confer resistance, HIV-1_YU2_ Env-pseudoviruses containing one or both of the N276D and K460N substitutions were tested for their sensitivity to 179NC75. All three of the virus mutants were found to be 179NC75-resistant (Fig 5E). We also tested the HIV_YU2_ N280Y, N332K, N160K and G459D virus mutants. As expected, and consistent with the ELISA data, the N280Y substitution conferred complete resistance to 179NC75, while the N332K and N160K changes had no effect. The G459D mutant was also 179NC75-sensitive (Fig 5E). We conclude, that 179NC75 is a potent neutralizing antibody that exerts selection pressure on HIV-1_YU2_ *in vivo* and drives the emergence of resistant viruses with sequence changes proximal to the CD4bs.

### Autologous viral selection by 179NC75

**Fig 6:**
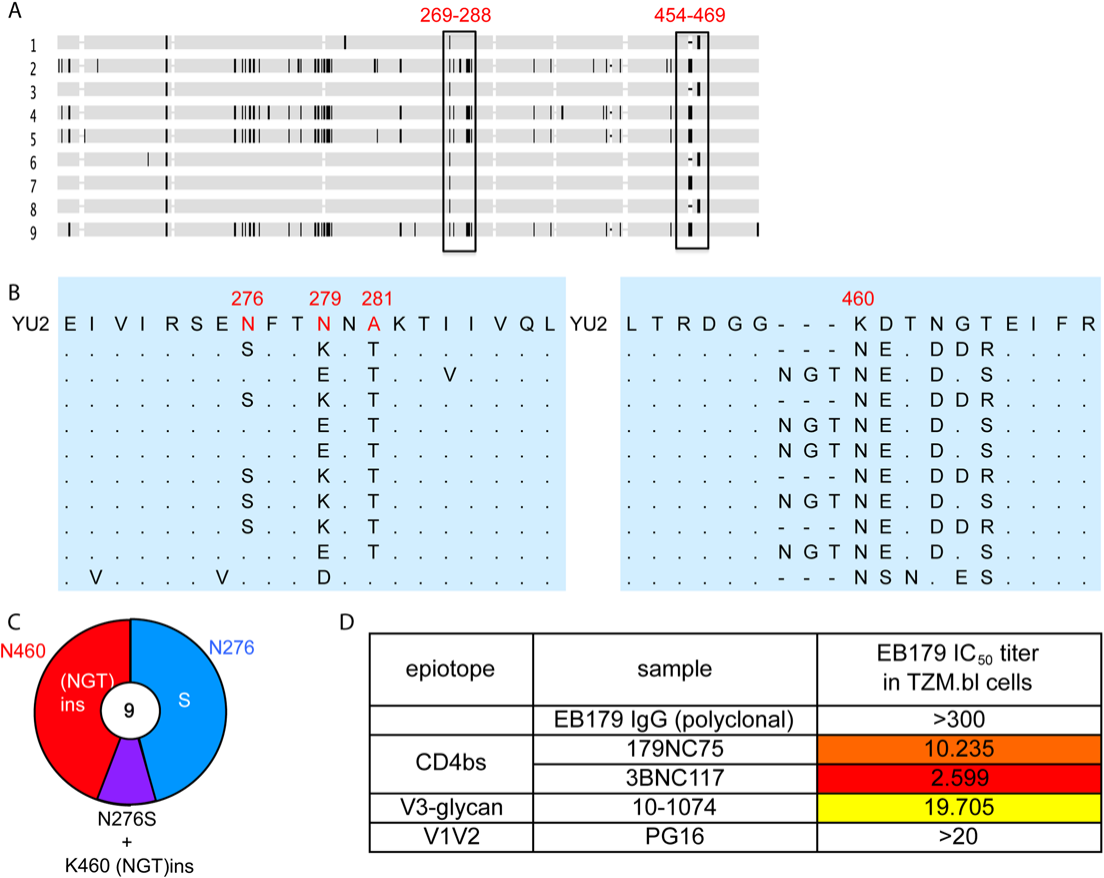
Autologous viruses from EB179. (A) Horizontal gray bars represents single gp120 sequences amplified and cloned from patient sera. The vertical rectangles show areas where recurrent mutations were found. (B) Expanded view of the most consistently mutated areas. (C) Pie chart comparing the recurrent mutations in gp120s from the EB179 autologous virus and HIV-1_YU2_. The number in the center of the pie denotes the total number of sequences cloned, and the sizes of slices are proportional to the number of sequences that carried the mutations; the color coding is same as used in Fig 5D. (D) The table shows IC_50_ values for the neutralization of the EB179 autologous culture virus by the autologous patient polyclonal IgG, as well as 179NC75, 3BNC117, 10–1074 and PG16 bNAbs.

To test whether 179NC75 exerted selective pressure on the autologous virus found in subject EB179, we cloned *env* genes from the donor’s T cells obtained at the time of the leukapheresis. All nine gp120 sequences obtained contained Asn at position 460, introducing a PNGS at that position in eight of the nine sequences (Fig 6A–6C). Five sequences contained an Asn-Gly-Thr insertion immediately N-terminal to position N460, resulting in the sequence NGTNET, and therefore adding another PNGS to the one that was already at position 460. Five other sequences contained the N276S mutation, eliminating the PNGS at position 276. One of the nine sequences included both the Asn-Gly-Thr insertion at position 460 and the N276S change (Fig 6C). Of note is that this pattern of sequence changes is highly similar to the escape mutations seen in the *env* genes of the 179NC75-treated, HIV-1_YU2_-infected hu-mice (Fig 5). To test whether the autologous virus from patient EB179 is resistant to 179NC75, we cultured the donor’s CD4+ T cells from the same leukapheresis sample that was used for the antibody isolation. Outgrown virus was then tested for neutralization in the TZM.bl assay for neutralization by the EB179 polyclonal IgG (from the same time point), as well as by 179NC75 and other known bNAbs including the CD4bs antibody 3BNC117 [7], the V3-stem binding antibody 10–1074 [42] and the V1/V2 apex-binding antibody PG16 [43] (Fig 6D). As expected, the EB179 polyclonal IgG failed to neutralize the autologous virus. Amongst the two CD4bs antibodies, the autologous virus was fourfold more resistant to 179NC75 than to 3BNC117, suggesting that the EB179 antibody repertoire has CD4bs antibodies that differ from 3BNC117 and VRC01-class bNAbs. Interestingly, the autologous virus was also resistant to PG16 and 10–1074, indicating that the patient may have additional neutralizing antibodies bearing similar specificities in his antibody repertoire. Taken together, the data imply that loop-based, glycan-dependent CD4bs bNAbs of the 179NC75 family exert selective pressure on HIV-1 *in vivo*.

## Discussion

The CD4bs is a highly conserved epitope on the HIV-1 Env and an important potential target for neutralizing antibodies. Although this site evolved to avoid antibody accessibility, two major groups of CD4bs bNAbs have been discovered [15]. The first group, exemplified by VRC01, is VH-restricted, IGVH1-2 or IGVH1-46, with the heavy chains positioned in a CD4-like orientation and CDRH2 making significant contacts with gp120 [6,7,15]. The CDRL3 [7,21,44], and in some cases also CDRL1 [6], of the corresponding light chains have to be short and compact to minimize potential interference and clashes with the glycans that surround the CD4bs. The emergence of these antibodies involves many somatic hypermutations, some of which are in the framework regions [45]. The second group of CD4bs bNAbs, which includes b12 and HJ16, is far more heterogeneous. These antibodies bind to gp120 via a CDRH3-dominated, loop based mechanism [15]. As might be expected, members of this group of CD4bs bNAbs arise from different VH segments and carry fewer somatic mutations [17–19]. The new antibody described in this study, 179NC75, is a loop binder that is closely related to HJ16. Similarly to HJ16, its Env-binding and virus-neutralizing activities are dependent on the N276 glycan [36]. Consistent with the CDRH3 loop-based mechanism of recognition that was described for antibodies that are not VH-restricted [15], when we generated the predicted germline version of 179NC75, where all mutations were reverted but the CDRH3 was retained, the antibody bound to BG505 SOSIP.664 trimers. This could indicate that any residual mutations present in the CDR3s of the reverted antibody might allow binding. Interestingly, the germline version of HJ16 also had some binding to BG505 SOSIP.664 trimers (Fig 4), however this binding was lower that the one of 179NC75, which could be attributed to a shorter CDRH3 (19 *versus* 24 residues).

Serum antibodies that are CD4bs-specific and N276-dependent have been described in HIV-1-infected individuals in two separate studies [32,46]. In the first study, an HJ16-type of CD4bs antibody response was found to be part of the second wave of serum neutralization in the CAP257 patient [46]. Viruses cloned from CAP257 after the emergence of these CD4bs antibodies carried an N276D or T278A mutation that were considered to be responses to antibody selection pressure [46]. In a second study, serum from individual VC1004 contained CD4bs-targeted NAbs that were sensitive to the N276D substitution but not D368R [47]. However, as the antibodies responsible for the serum activity were not cloned in either study much of what we know about the *in vivo* activity of these N276-dependent class of CD4bs antibodies is inferential. Our 179NC75 therapy experiments in HIV-1–infected hu-mice demonstrate that escape variants contain very similar, and sometimes identical, mutations to ones present in the autologous virus isolated from the infected human from whom the 179NC75 antibody was also derived. We conclude that the CDRH3-dominted N276-dependent CD4bs antibodies are effective at suppressing viremia *in vivo* and thence driving the emergence of escape variants.

## Supporting Information

S1 Fig Number of mutations in V-region in the 2CC core-sorted antibodies. The number of nucleotide mutations in sequences that are part of a clonal family (“clonally related”) is compared to sequences that appeared only once (“singles”). (B) The same analysis as in (A), but for the light chains.
(PDF)

S2 Fig Binding of 179NC75 and its variants to soluble Env proteins in ELISA. Each of the four variants, 179NC21, 65, 75 and 1055, was tested for binding to 2CC core, monomeric gp120 and gp140 foldon proteins. VRC01 and PGT121 were used as controls.
(PDF)

S3 Fig SPR binding studies. The upper panel shows sensograms from SPR binding studies. BG505 SOSIP.664 trimers expressed in HEK203-6E cells (resulting in a mixture of high-mannose and complex N-glycans) or HEK203-6E cells treated with kifunensine (resulting in high-mannose N-glycans only) were immobilized by injecting them over the indicated capture antibodies. 179NC75 Fab was then injected over captured trimers as a 4-fold dilution series with a top concentration of 500 nM. Residuals for a 1:1 binding model fit to the sensorgram data are shown below each sensogram from which *K*_D_ values were obtained (see table in the lower panel of the figure). The weak binding responses to high-mannose-only BG505 SOSIP.664 trimers (bottom panel) could not be fit to a binding model. The table in the lower panel summerizes the affinities of 179NC75 Fab for BG505 SOSIP.664 derived by surface plasmon resonance (SPR). On/off rates (*k*_a_/*k*_d_) and binding constants (*K*_D_ (M)) were calculated by kinetic analyses after subtraction of backgrounds using a 1:1 binding model using the Biacore T200 Evaluation software.
(PDF)

S4 Fig Binding of 179NC75 and its variants to BG505 SOSIP,664-D7324 trimers in ELISA. Each of the four variants, 179NC21, 65, 75 and 1055, was tested for their binding to BG505 SOSIP.664-D7324 trimers. The quaternary structure-influenced bNAbs PGT145 and PG16 [43] served as positive controls, and MGO.53 as a negative control.
(PDF)

S1 Table IC_50_ values for purified IgG from EB179 tested in TZM.bl assays. **(A)** Neutralization of a panel of 14 cross-clade pseudoviruses. The shades of red and yellow indicate the IC_50_ and IC_80_ concentrations, with red representing the lowest IgG concentration at which a virus was neutralized and yellow the highest concentration. The white color denotes that the virus was not neutralized at any antibody concentration tested. **(B**) Neutralization IC_50_ values for purified IgG from EB179 against a panel of HIV-1_YU2_ mutant viruses. The color-coding scheme is the same as in (A).
(XLSX)

S2 Table Expressed antibodies from 2CC core-sorted B cells. The V(D)J segments of the heavy and light chains for each expressed antibody are representative of the different 2CC core-reactive IgG B cell clones. The colors of the different rows in the table correspond to the colors used on the pie chart shown in Fig 1A. The numbers of nucleotide mutations in Vh and Vl, as well as the CDR3 sequences and lengths (i.e., number of amino acids), are indicated in the table.
(XLSX)

S3 Table Neutralizing activity of 179NC75. The antibody was tested against an extended panel of Tier-2 viruses. The color scheme is the same as used in S1 Table.
(XLSX)

S4 Table Summary of IC_50_ and IC_80_ values for the 120 virus test panel. The number of viruses tested for each clade and the % of breadth (i.e., the proportion neutralized) is shown. The mean IC_50_ and IC_80_ values represent a geometric mean of all the IC_50_ and IC_80_ values derived for the given clade. The total % breadth and IC_50_ and IC_80_ values were calculated by averaging the corresponding values for each individual clade. The color-coding scheme is the same as used in S1 Table.
(XLSX)

S5 Table IC_50_ values of 179NC75, 3BNC117, VRC01 and HJ16 tested against 53 common viruses. A comparison between the IC_50_ values in TZM.bl assay of 179NC75, 3BNC117 [7], VRC01 [7] and HJ16 [49]. The color scheme is the same as used in S1 Table.
(XLSX)

S6 Table Comparison of 179NC75, 3BNC60 and 8ANC195 binding to BG505 SOSIP.664 containing a mixture of complex and high-mannose or exclusively high-mannose glycans. PGT145, 35O22 or PGT121 IgG were used to capture BG505 SOSIP.664 produced in HEK293-6E cells (resulting in a mixture of complex and high-mannose glycans) or HEK293-6E cells treated with kifunensine (resulting in high-mannose glycans only). Due to different binding efficiencies of each capture antibody, final trimer capture levels differed as indicated. Fab fragments of 179NC75, 3BNC60 and 8ANC195 were injected over the resulting surfaces at 500 nM concentration, resulting in the indicated binding levels. Comparing the ratios of binding levels for Fabs to both glycoforms of captured trimer demonstrates that 179NC75 binds preferentially to BG505 SOSIP.664 trimer containing mixed (complex and high-mannose) glycans, while the control antibodies 3BNC60 and 8ANC195 are not sensitive to the trimer glycoform.
(XLSX)
