# Supplementary figures and images for: A New Glycan-Dependent CD4-Binding Site Neutralizing Antibody Exerts Pressure on HIV-1 In Vivo
Source: PLoS Pathog. 2015 Oct 30;11(10):e1005238. doi: 10.1371/journal.ppat.1005238 (PMC4627763; doi:10.1371/journal.ppat.1005238)

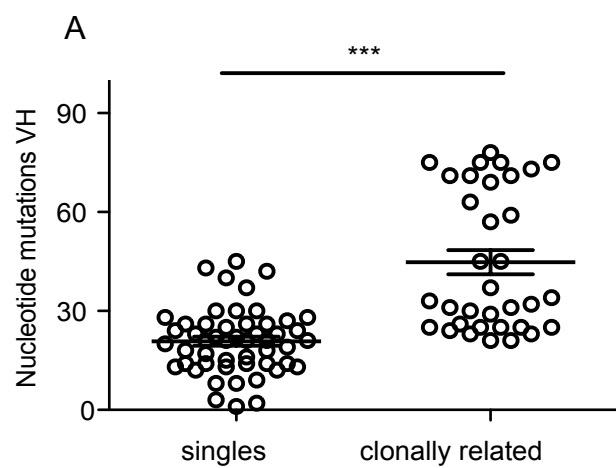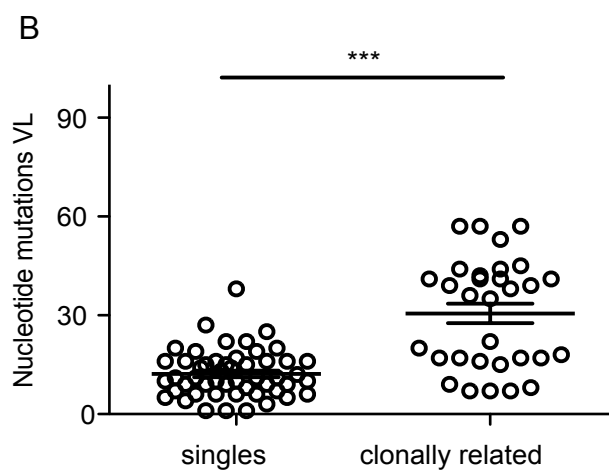

Supplement: S1 Fig — The number of nucleotide mutations in sequences that are part of a clonal family (“clonally related”) is compared to sequences that appeared only once (“singles”). (B) The same analysis as in (A), but for the light chains. (PDF) [file ppat.1005238.s001.pdf]

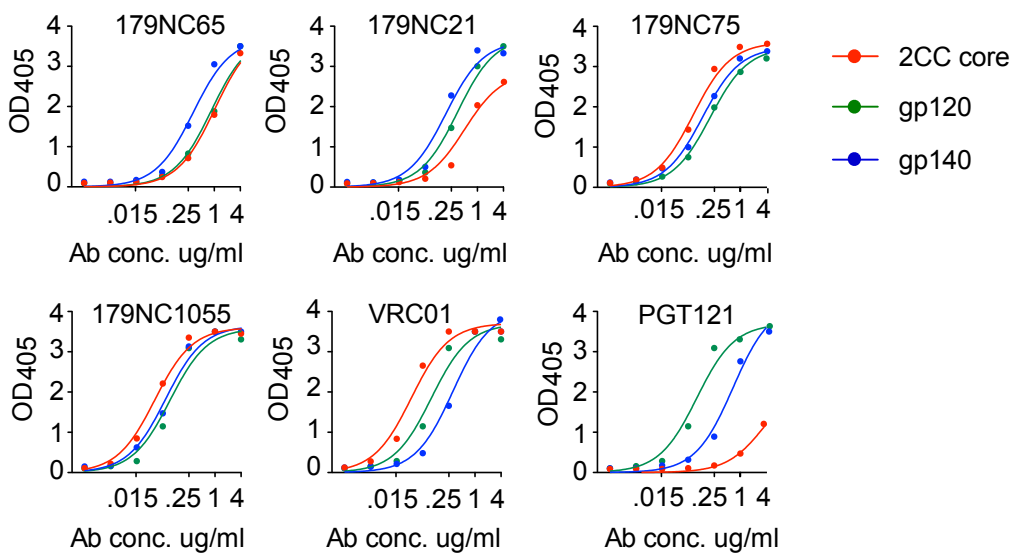

Supplement: S2 Fig — Each of the four variants, 179NC21, 65, 75 and 1055, was tested for binding to 2CC core, monomeric gp120 and gp140 foldon proteins. VRC01 and PGT121 were used as controls. (PDF) [file ppat.1005238.s002.pdf]

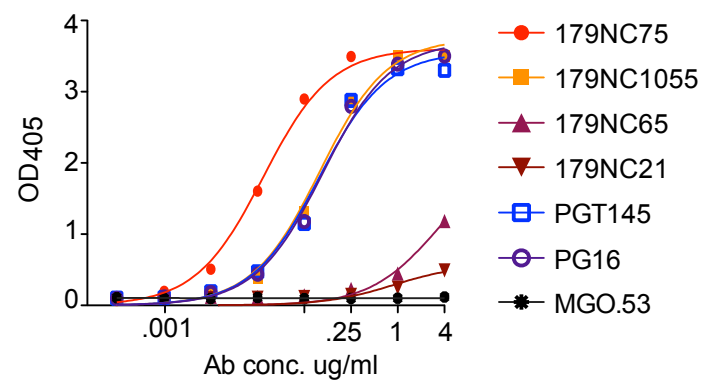

Supplement: S4 Fig — Each of the four variants, 179NC21, 65, 75 and 1055, was tested for their binding to BG505 SOSIP.664-D7324 trimers. The quaternary structure-influenced bNAbs PGT145 and PG16 [43] served as positive controls, and MGO.53 as a negative control. (PDF) [file ppat.1005238.s004.pdf]
