# Supplementary material for: A New Glycan-Dependent CD4-Binding Site Neutralizing Antibody Exerts Pressure on HIV-1 In Vivo
Source: PLoS Pathog. 2015 Oct 30;11(10):e1005238. doi: 10.1371/journal.ppat.1005238 (PMC4627763; doi:10.1371/journal.ppat.1005238)
Supplement: S3 Fig — The upper panel shows sensograms from SPR binding studies. BG505 SOSIP.664 trimers expressed in HEK203-6E cells (resulting in a mixture of high-mannose and complex N-glycans) or HEK203-6E cells treated with kifunensine (resulting in high-mannose N-glycans only) were immobilized by injecting them over the indicated capture antibodies. 179NC75 Fab was then injected over captured trimers as a 4-fold dilution series with a top concentration of 500 nM. Residuals for a 1:1 binding model fit to the sensorgram data are shown below each sensogram from which K D values were obtained (see table in the lower panel of the figure). The weak binding responses to high-mannose-only BG505 SOSIP.664 trimers (bottom panel) could not be fit to a binding model. The table in the lower panel summerizes the affinities of 179NC75 Fab for BG505 SOSIP.664 derived by surface plasmon resonance (SPR). On/off rates (k a/k d) and binding constants (K D (M)) were calculated by kinetic analyses after subtraction of backgrounds using a 1:1 binding model using the Biacore T200 Evaluation software. (PDF) [file ppat.1005238.s003.pdf]

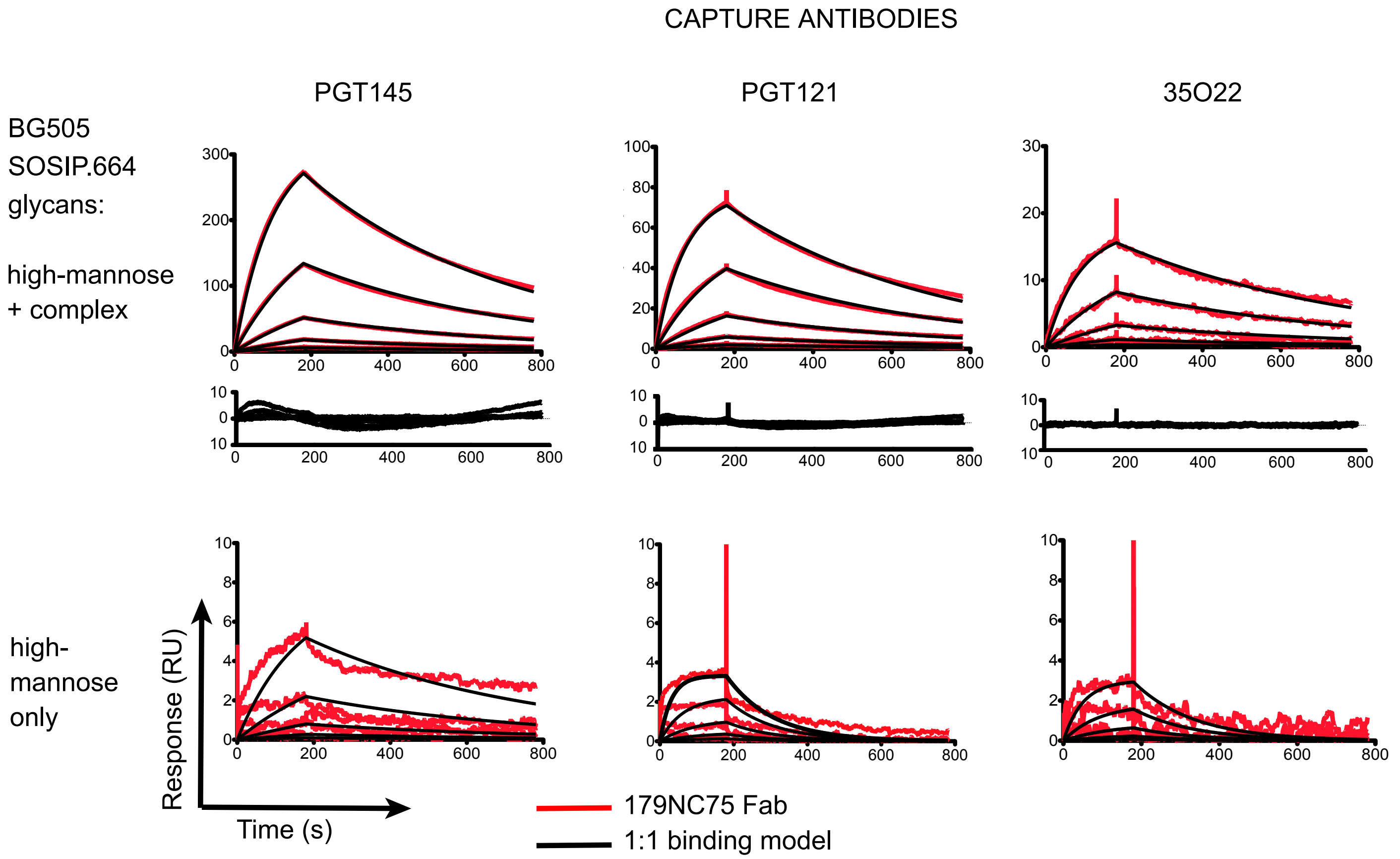

Analyte	Capture antibody/ligand	k_a (1/Ms)	k_d (1/s)	K_D (M)	Rmax (RU)	Chi² (RU²)
179nc75 Fab	PGT121 IgG	1.92E+04	1.84E-03	9.61E-08	104.1	1.36
179nc75 Fab	PGT145 IgG	1.59E+04	1.87E-03	1.18E-07	402.4	5.54
179nc75 Fab	35O22 IgG	1.61E+04	1.45E-03	9.00E-08	44	0.185
